# Immunogenicity and Safety of the HZ/su Adjuvanted Herpes Zoster Subunit Vaccine in Adults Previously Vaccinated With a Live Attenuated Herpes Zoster Vaccine

**DOI:** 10.1093/infdis/jix482

**Published:** 2017-09-20

**Authors:** Katrijn Grupping, Laura Campora, Martine Douha, Thomas C Heineman, Nicola P Klein, Himal Lal, James Peterson, Ilse Vastiau, Lidia Oostvogels

**Affiliations:** 1 GSK Vaccine, Wavre, Belgium; 2 Genocea Biosciences, Cambridge, Massachusetts; 3 Kaiser Permanente Vaccine Study Center, Oakland, California; 4 Pfizer Inc, Collegeville, Pennsylvania; 5 Foothill Family Clinic, Salt Lake City, Utah

**Keywords:** herpes zoster, HZ/su adjuvanted herpes zoster subunit vaccine, live attenuated zoster vaccine Zostavax, immune response, revaccination

## Abstract

**Background:**

Protection against herpes zoster (HZ) induced by the live attenuated zoster vaccine Zostavax (ZVL) wanes within 3–7 years. Revaccination may renew protection. We assessed whether (re)vaccination with the adjuvanted HZ subunit vaccine candidate (HZ/su) induced comparable immune responses in previous ZVL recipients and ZVL-naive individuals (HZ-NonVac).

**Methods:**

In an open-label, multicenter study, adults ≥65 years of age, vaccinated with ZVL ≥5 years previously (HZ-PreVac), were matched to ZVL-naive adults (HZ-NonVac). Participants received 2 doses of HZ/su 2 months apart. The primary objective of noninferiority of the humoral immune response 1 month post–dose 2 was considered demonstrated if the upper limit of the 95% confidence interval (CI) of the adjusted anti–glycoprotein E geometric mean concentration (GMC) ratio of HZ-NonVac over HZ-PreVac was <1.5. HZ/su cellular immunogenicity, reactogenicity, and safety were also assessed.

**Results:**

In 430 participants, humoral immune response to HZ/su was noninferior in HZ-PreVac compared with HZ-NonVac (adjusted GMC ratio, 1.04 [95% CI, .92–1.17]). Cellular immunogenicity, reactogenicity, and safety appeared to be comparable between groups. HZ/su was well-tolerated, with no safety concerns raised within 1 month post–dose 2.

**Conclusions:**

HZ/su induces a strong immune response irrespective of prior vaccination with ZVL, and may be an attractive option to revaccinate prior ZVL recipients.

**Clinical Trials Registration:**

NCT02581410.

## Teaser

The adjuvanted herpes zoster subunit vaccine candidate induces strong humoral and cellular immune responses irrespective of prior vaccination with the live attenuated zoster vaccine (ZVL), and may be an attractive option to revaccinate prior ZVL recipients.

**(See the major article Schwarz et al, on pages 1352–61 and editorial commentary by Oxman et al, on pages 1329–33.)**

Herpes zoster (HZ) results from reactivation of latent varicella zoster virus (VZV) and usually presents as a vesicular dermatomal rash [1]. HZ can be followed by postherpetic neuralgia (PHN), chronic neuropathic pain that persists after resolution of the zoster rash [2]. A decline in VZV-specific cell-mediated immunity (CMI) increases the risk of HZ [3–5]. Because CMI naturally decreases with age [6], the risk of developing HZ, and the risk of PHN, increases as people get older. The incidence of HZ increases from <5 cases per 1000 person-years (PY) in those <50 years of age to 4–8 cases per 1000 PY in adults between 50 and 59 and >10 cases per 1000 PY in adults >70 years of age [7, 8]. Half of all HZ cases occur in adults aged >60 years, and the lifetime risk is 50% in those surviving to 85 years of age [9]. The risk of developing PHN increases from 5% to 14% in HZ patients aged 50–59 years to >15% in those >70 years of age [10, 11].

Many HZ complications, but not PHN, can be prevented with antivirals if administered shortly after disease onset [1, 12]. Vaccination of at-risk populations is a cost-effective approach to prevent HZ and its complications [13]. The live attenuated zoster vaccine (ZVL) licensed for use in healthy adults >50 years of age (Zostavax, a trademark of Merck Sharp & Dohme Corporation) reduces HZ incidence by 70% in people between 50 and 59 years of age [14]. In the United States, vaccination is currently recommended to prevent HZ in people >60 years of age [9] and the efficacy of ZVL in this population is lower: 51% in people >60 years and 38% in people >70 years of age [15]. In adults >60 years of age, efficacy against HZ declines to 30.6% in the sixth year [16] and to 21.1 % between 7 and 11 years postvaccination [17]. The vaccine efficacy against PHN wanes from 66.5% shortly after vaccination [15] to 35.4% between 7 and 11 years postvaccination [17].

HZ/su is a subunit vaccine candidate that contains the recombinant VZV glycoprotein E (gE) [18, 19], adjuvanted with the proprietary AS01 Adjuvant System (GSK Vaccines) [20, 21]. HZ/su substantially boosts the immune response to gE [22–24]. Interestingly, the humoral immune response to the vaccine does not markedly differ between people aged 50–59 years and those >70 years of age [23], and both the humoral and cellular immune responses persist above baseline levels for at least 6 years postvaccination [25]. HZ/su has shown an efficacy of >90% for the prevention of HZ in people 50 years of age and older [26, 27]. Moreover, vaccine efficacy against HZ persisted for at least 4 years after vaccination, with 88% efficacy in the fourth year postvaccination [27].

ZVL continues to provide protection against the incidence of HZ through the first 5 years after vaccination [16], and revaccination after year 5 may therefore be beneficial [28]. A second vaccination with ZVL in previously vaccinated individuals 10 years after the initial vaccination induces a VZV-specific CMI response higher than in age-matched controls who had never been vaccinated with ZVL, showing that immune responses can be boosted by a second dose [29]. However, given HZ/su’s high vaccine efficacy against HZ and PHN across all age groups, revaccinating older adults, who were previously vaccinated with ZVL, with HZ/su may be an attractive alternative to reduce the risk of HZ and PHN. This study therefore compared immunogenicity and assessed reactogenicity and safety of HZ/su in adults aged ≥65 years who were vaccinated with ZVL ≥5 years before study start and group-matched ZVL-naive adults.

## METHODS

### Study Design and Participants

This study is a phase 3, open-label, group-matched, multicenter study conducted in the United States. Adults ≥65 years of age who were previously vaccinated with ZVL (Zostavax) ≥5 years prior to study start (HZ-PreVac) and group-matched ZVL-naive adults (HZ-NonVac) were enrolled (for the distribution of matching criteria at vaccination, see Supplementary Table 1). The active phase of the study started in March 2016 (first vaccination) and was completed in August 2016 (1 month post–dose 2). The extended safety follow-up is expected to be completed in August 2017.

Participants in the HZ-NonVac group were group-matched to those in the HZ-PreVac group according to the predefined variables age (65–69, 70–79, ≥80 years), sex, race (white, African American, Hispanic, and other), and medical condition. Medical conditions were ranked in a hierarchical order (immune-mediated diseases, diabetes mellitus, current depression, pulmonary disorders, heart conditions, none of these medical conditions), and participants were matched according to the highest-ranked condition.

Study participants were men or women aged ≥65 years at the time of the first vaccination with HZ/su. Adults eligible for inclusion in the HZ-PreVac group had received ZVL at least 5 years prior to study start. Participants provided written informed consent before study start. Adults were excluded from participation if they had received or were scheduled to receive a live vaccine within 30 days, had received any investigational or nonregistered drug or vaccine within 30 days, had received immunosuppressants or other immune-modifying drugs for >14 consecutive days within 180 days, or had received any long-acting immune-modifying drugs within 180 days before the first HZ/su vaccination. Adults with a history of HZ, or adults scheduled to receive a HZ vaccine other than HZ/su, as well as adults with a history of any reaction or hypersensitivity to any of the vaccine components, were excluded from participation.

The study protocol was reviewed and approved by the institutional review boards (Chesapeake institutional review board [IRB], Columbia, MD; Office of Human Research IRB, Philadelphia, PA; Kaiser Permanente, Northern California IRB, Oakland, CA; Marshfield Clinic Research Foundation IRB, Marshfield, WI). The study was conducted in accordance with the Declaration of Helsinki and the principles of Good Clinical Practice, and is registered at ClinicalTrials.gov (NCT02581410) and available at http://www.gsk- clinicalstudyregister.com (study ID: 201198).

### Study Vaccines

Study participants in both groups received 2 intramuscular doses of HZ/su 2 months apart. Participants were vaccinated in the deltoid region of the nondominant arm. Each dose of HZ/su contained 50 μg of the gE antigen and the GSK proprietary AS01_B_ Adjuvant System (containing 50 μg of 3-O-desacyl-4’-monophosphoryl lipid, 50 μg of *Quillaja saponaria* Molina, fraction 21 [QS21, Licensed by GSK from Antigenics LLC, a wholly owned subsidiary of Agenus Inc], and liposome).

### Study Objectives and Measures

#### Study Objectives

The co-primary objectives of the study were to compare the humoral immune responses 1 month after dose 2 of HZ/su between the HZ-PreVac and HZ-NonVac groups, and to evaluate safety and reactogenicity up to 1 month after dose 2 of HZ/su in both study groups. The secondary study objectives also presented in this manuscript were to assess the humoral and CMI responses to the HZ/su vaccine at baseline (prevaccination), and 1 month post–dose 1 and post–dose 2 in both study groups.

#### Assessment of Immunogenicity

**Figure 1.**
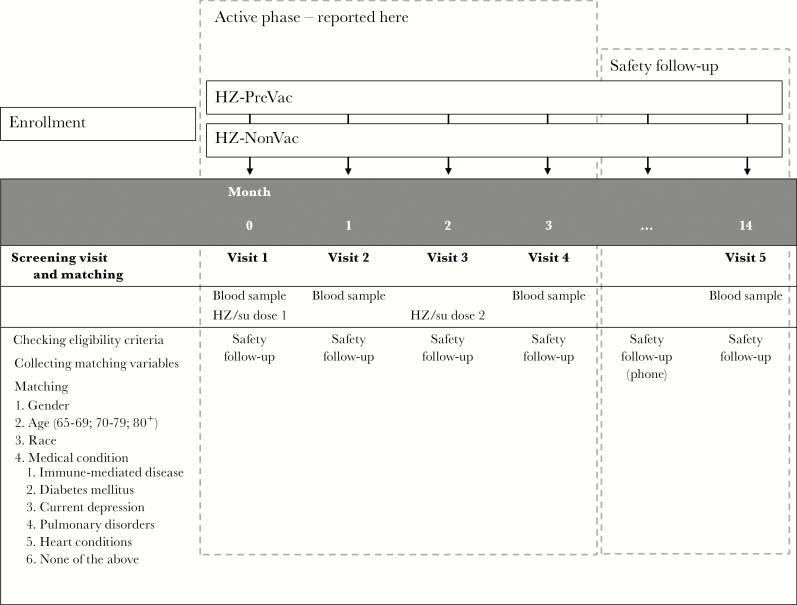
Study design. Before the first participant was vaccinated, potential participants were screened for eligibility and matching purposes. Matched participants were included in the study. During the active phase of the study, participants visited the study center at specified timepoints for a blood draw to determine immune parameters (months 0, 1, and 3), and to receive the study vaccine (months 0 and 2). Only data collected during the active phase of the study are reported in this manuscript. The safety follow-up was expected to continue until August 2017. During this safety follow-up, participants are being followed for safety through monthly phone calls. A final blood draw is scheduled to take place at 12 months after the second dose of study vaccine. Abbreviations: HZ-NonVac, participants who never received the live attenuated zoster vaccine; HZ-PreVac, participants who received the live attenuated zoster vaccine ≥5 years prior to study start; HZ/su, herpes zoster subunit candidate vaccine.

Blood samples for the immunogenicity assessments were collected at baseline and at 1 month after the first and second vaccine doses (Figure 1). Anti-gE antibody concentrations were measured by anti-gE enzyme-linked immunosorbent assay. The assay cutoff was 97 mIU/mL. CMI responses were assessed by intracellular cytokine staining and flow cytometry, as detailed previously [25]. In brief, peripheral blood mononuclear cells were stimulated in vitro with gE peptides, after which frequencies of gE-specific CD4^+^ T cells expressing at least 2 activation markers (here referred to as CD4^2+^) of the 4 markers assessed (interferon-γ, interleukin 2, tumor necrosis factor–α, and CD40 ligand) were determined.

#### Assessment of Safety

Solicited adverse events (AEs) were reported on diary cards provided to study participants and recorded for 7 days (days 0–6) after each vaccination. Solicited AEs were recorded as local (injection site pain, redness, and swelling) or systemic (fatigue, fever, gastrointestinal symptoms, headache, myalgia, and shivering). Unsolicited AEs were recorded for 30 days after each vaccination, and included any AE not recorded as a solicited AE. The intensity of all AEs was graded on a scale of 1 to 3. A grade 3 (severe) unsolicited AE was defined as preventing normal activities. Solicited AEs were defined as grade 3 when preventing normal everyday activity (for pain, headache, fatigue, gastrointestinal symptoms, myalgia, shivering), when presenting a surface diameter >100 mm (for redness and swelling), or when presenting as a tympanic/oral/axillary temperature >39.0°C (for fever). All solicited local AEs were considered causally related to vaccination. The causality of all other AEs was assessed by the investigator.

Serious adverse events (SAEs) and potential immune-mediated diseases (pIMDs) were recorded for the entire duration of the study, but only findings from the active phase of the study (first vaccination visit through 30 days post–dose 2) are presented here. A full list of pIMDs is provided in Supplementary Table 2.

### Statistical Analyses

All statistical analyses were performed using the SAS software version 9.3 TS1M2 on Windows SDD 4.3.3.

Immunogenicity data were analyzed on the according to protocol cohort, which included all participants who complied with protocol-specified procedures and for whom data were available. For inferential analyses of the co-primary endpoint data, an analysis of variance (ANOVA) model was used on log-transformed antibody concentration data and included the vaccine group and the group-matching categories as fixed effects. Adjusted means and a difference of means between both study groups were calculated together with 2-sided confidence intervals (CIs) and back-transformed to the original units to provide adjusted geometric mean concentrations (GMCs) and GMC ratio. Per protocol, noninferiority of the response was demonstrated if the upper limit of the 2-sided CI of the adjusted GMC ratio of the HZ-NonVac over the HZ-PreVac group at 1 month post–dose 2 (active phase) was <1.5. Secondary immunogenicity endpoint data, including CMI data presented here, were evaluated using descriptive analyses. For descriptive humoral immunogenicity data, the 95% CI for GMCs was obtained for each group separately. First, a 95% CI for the mean of log-transformed concentrations was obtained, under the assumption that log-transformed values were normally distributed with unknown variance. Subsequently, the 95% CI for GMCs was calculated by anti-log transformation of the previously calculated 95% CI for the mean of log-transformed concentrations. For descriptive cellular immunogenicity, the frequency of gE-specific CD4^2+^ T cells was calculated as the difference between the frequency of CD4^2+^ T cells, stimulated in vitro with the gE antigen and those stimulated with culture alone. Descriptive statistics (min, Q1, median, Q3, max) of CD4^2+^ T cells were tabulated by group at all timepoints.

Safety and reactogenicity data were evaluated using descriptive analyses. Safety data were analyzed in the total vaccinated cohort of participants who received at least 1 dose of HZ/su.

Based on variability in the anti-gE antibody response to HZ/su as seen in previous clinical trials, a sample size of 190 evaluable participants per study group would demonstrate noninferiority in humoral immunogenicity with at least 99% power.

## RESULTS

### Participants

**Figure 2.**
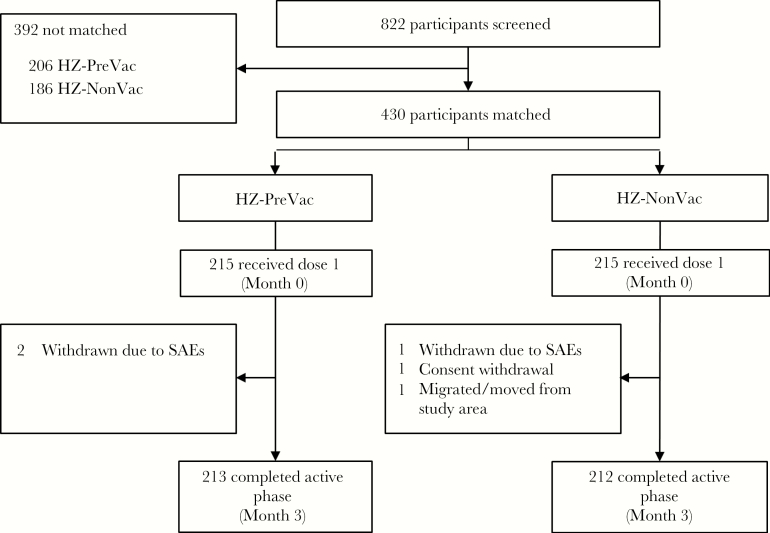
Participant disposition. Potential participants were first screened and matching variables were collected (see Figure 1). Only matched participants were vaccinated with herpes zoster subunit candidate vaccine (see Supplementary Table 1 for additional information on matching). Abbreviations: HZ-NonVac, participants who never received the live attenuated zoster vaccine; HZ-PreVac, participants who received live attenuated zoster vaccine ≥5 years prior to study start; SAE, serious adverse event.

A total of 822 older adults were screened for participation in this study. Of these, 215 people not previously vaccinated were matched according to prespecified criteria (age, sex, geographic ancestry, and medical condition) to 215 people who had previously been vaccinated with ZVL (Figure 2). Of the 430 vaccinated participants, 425 (98.8%) completed the active phase of the study. Demographic characteristics were comparable for participants in both study groups and are presented in Table 1. Details of participant matching are provided in Supplementary Table 1.

**Table 1.** Characteristics of Study Participants (Total Vaccinated Cohort)

Characteristic	Total (N = 430)	HZ-NonVac (n = 215)	HZ-PreVac (n = 215)
Age, mean (SD)	70.9 (4.6)	70.8 (4.6)	71.1 (4.5)
Sex, No (%)			
Female	220 (51.2)	111 (51.6)	109 (50.7)
Male	210 (48.8)	104 (48.4)	106 (49.3)
White/European ancestry, No. (%)	430 (100)	215 (100)	215 (100)

Abbreviations: HZ-NonVac, participants who never received the live attenuated zoster vaccine; HZ-PreVac, participants who received the live attenuated zoster vaccine ≥5 years prior to study start; N/n, number of participants; SD, standard deviation.

### Immunogenicity

**Figure 3.**
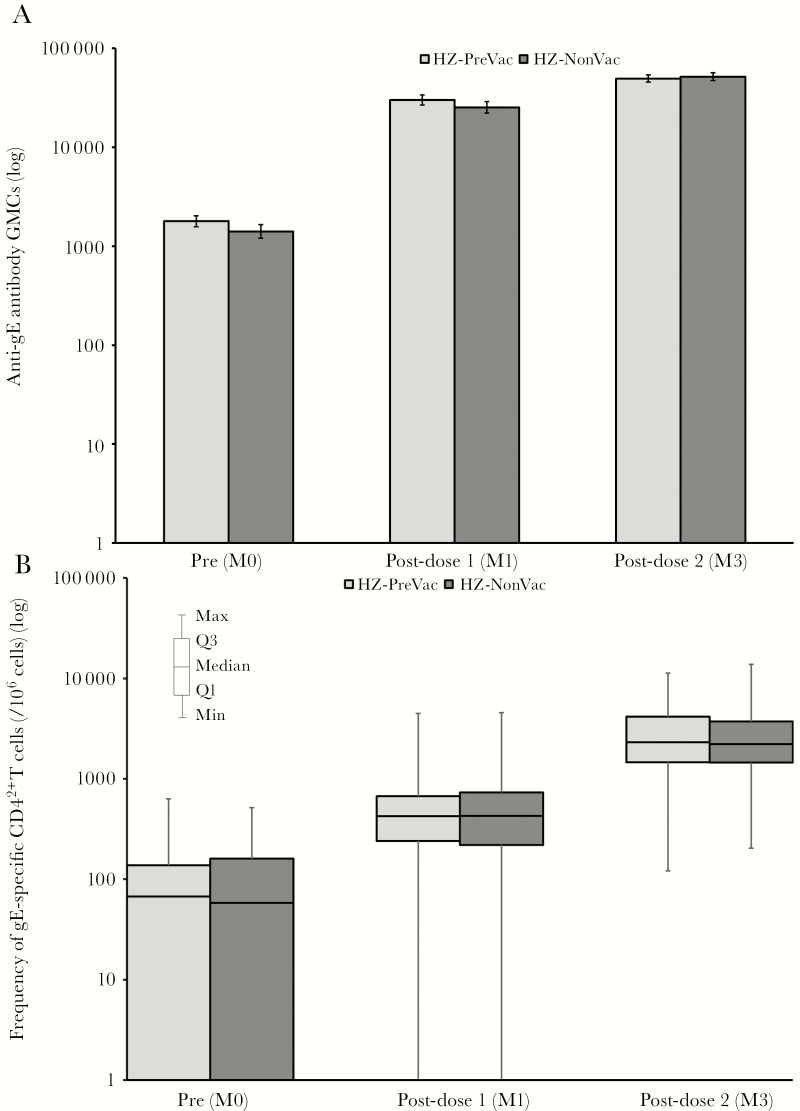
Humoral and cellular immune response to herpes zoster subunit candidate vaccine (HZ/su) (according to protocol cohort for immunogenicity). *A*, Humoral immune response to HZ/su vaccination. Anti–glycoprotein E (gE) antibody concentrations were determined by enzyme-linked immunosorbent assay. Data are geometric mean concentrations (GMCs [mIU/mL]) and error bars indicate 95% confidence intervals. *B*, Cellular immune response to HZ/su vaccination. The gE-specific CD4^+^ cells expressing at least 2 activation markers (CD4^2+^) were determined by intracellular staining and flow cytometry. Data are median cell counts per 10^6^ total peripheral blood mononuclear cells. Light bars indicate participants who received the live attenuated zoster vaccine ≥5 years prior to study start (HZ-PreVac group); dark bars indicate participants who never received the live attenuated zoster vaccine (HZ-NonVac group). Abbreviations: M0 = pre vaccination; M1, one month post-dose 1; M3, one month post-dose 2.

Prior to the first vaccination, all evaluable participants in the HZ-PreVac group and 98% of evaluable participants in the HZ-NonVac group were seropositive for anti-gE antibodies (anti-gE concentration above the assay cutoff of 97 mIU/mL). Anti-gE antibody GMCs appeared similar at baseline in both study groups and increased markedly after both vaccine doses (Figure 3A and Supplementary Table 3). Anti-gE antibody GMCs post–dose 2 were comparable for both study groups, with an adjusted GMC ratio of 1.04 (Table 2). The primary immunologic study objective was met, as the upper limit of the adjusted GMC ratio of the HZ-NonVac group over the HZ-PreVac group was below the 1.5 cutoff (Table 2).

**Table 2.** Adjusted Geometric Mean Concentrations (GMCs) and Adjusted GMC Ratio of Anti–Glycoprotein E Antibody Concentrations 1 Month Post–Dose 2 (According to Protocol Cohort for Immunogenicity)

HZ-NonVac				HZ-PreVac				GMC Ratio (HZ-NonVac/HZ-PreVac)		
		95% CI^a^				95% CI^a^			95% CI^b^	
No.	Adjusted GMC	Lower Limit	Upper Limit	No.	Adjusted GMC	Lower Limit	Upper Limit	Value	Lower Limit	Upper Limit
204	50522.9	44347.4	57558.4	204	48589.4	42649.4	55356.6	1.04	0.92	1.17^c^

Abbreviations: adjusted GMC, geometric mean antibody concentration adjusted for group-matching variable; CI, confidence interval; HZ-NonVac, participants who never received the live attenuated zoster vaccine; HZ-PreVac, participants who received live attenuated zoster vaccine ≥5 years prior to study start; No., number of participants with both pre- and postvaccination results available.

^a^95% confidence intervals for the adjusted GMC (analysis of variance [ANOVA] model: adjustment for group-matching variable) – pooled variance.

^b^95% confidence interval for the adjusted GMC ratio (ANOVA model: adjustment for group-matching variable) – pooled variance.

^c^Primary objective considered met if <1.5.

At baseline, the median CD4^2+^ T-cell frequency appeared similar in both groups. After dose 1, median frequencies of gE-specific CD4^2+^ T cells increased in both groups, and a more substantial overall increase was seen after dose 2 (Figure 3B and Supplementary Table 3). No difference in CD4^2+^ T-cell frequency was apparent between study groups.

### Reactogenicity and Safety

**Figure 4.**
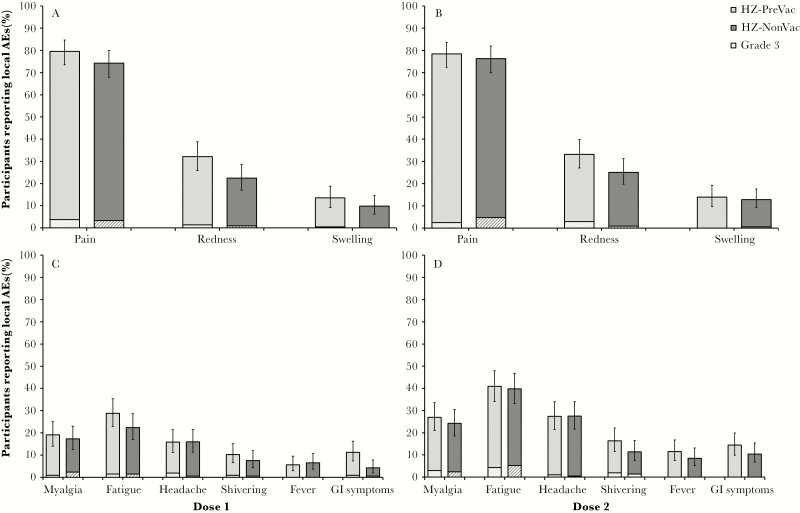
Solicited adverse events (AEs) after vaccination doses (total vaccinated cohort). *A*, Percentage of participants reporting local solicited AEs after herpes zoster subunit candidate vaccine (HZ/su) dose 1. *B*, Percentage of participants reporting local solicited AEs after HZ/su dose 2. *C*, Percentage of participants reporting related systemic solicited AEs after HZ/su dose 1. *D*, Percentage of participants reporting related systemic solicited AEs after HZ/su dose 2. Light bars indicate participants who received the live attenuated zoster vaccine ≥5 years prior to study start (HZ-PreVac group); dark bars indicate participants who never received the live attenuated zoster vaccine (HZ-NonVac group). Striped sections indicate grade 3 solicited AEs. Error bars indicate 95% confidence intervals.

The percentage of participants reporting all-grade solicited local and systemic AEs, as well as grade 3 solicited AEs, was comparable between study groups (Table 3). The most common local solicited AE reported after each HZ/su dose was pain. The most common systemic solicited AE reported after each dose was fatigue (Figure 4). Solicited AEs were transient with a median duration of ≤3 days for local AEs and ≤2 days for systemic AEs.

**Table 3.** Reactogenicity and Safety After Vaccination With Adjuvanted Herpes Zoster Subunit Candidate Vaccine (Total Vaccinated Cohort)

Adverse Event	HZ-NonVac (n = 214)		HZ-PreVac (n = 215)	
	No.^a^	% (95% CI)	No.^a^	% (95% CI)
Solicited AEs				
Within the 7-day (days 0–6) postvaccination period				
Participants reporting any solicited local reaction	187	87.4 (82.2–91.5)	193	89.8 (84.9–93.5)
Grade 3 solicited local reactions	21	9.8 (6.2–14.6)	21	9.8 (6.1–14.5)
Participants reporting any solicited systemic reaction	154	72.0 (65.4–77.9)	149	69.3 (62.7–75.4)
Grade 3 solicited systemic reactions	24	11.2 (7.3–16.2)	23	10.7 (6.9–15.6)
	HZ-NonVac (n = 215^b^)		HZ-PreVac (n = 215^b^)	
Unsolicited AEs				
Within the 30-day (days 0–29) postvaccination period				
Total reported unsolicited AEs	83	—	125	—
Participants reporting any unsolicited AE	52	24.2 (18.6–30.5)	78	36.3 (29.8–43.1)
Unsolicited AEs considered related by investigator	12	5.6 (2.9–9.5)	13	6.0 (3.3–10.1)
Grade 3 unsolicited AEs	5	2.3 (.8–5.3)	14	6.5 (3.6–10.7)
SAEs				
From the first vaccination up to 30 days after last vaccination				
Total reported SAEs	4	—	5	—
Participants reporting any SAE	4	1.9 (.5–4.7)	4	1.9 (.5–4.7)
SAEs considered related by investigator	0		0	
pIMDs				
From the first vaccination up to 30 days after last vaccination				
Total reported pIMDs	0	—	0	—

Abbreviations: AE, adverse event; CI, exact 2-sided confidence interval; HZ-NonVac, participants who never received the live attenuated zoster vaccine; HZ-PreVac, participants who received the live attenuated zoster vaccine ≥5 years prior to study start; n, number of participants with at least 1 administered dose and solicited adverse event symptom screen completed; pIMD, potential immune-mediated disease; SAE, serious adverse event.

^a^Total number of AEs, SAEs, pIMDs, or participants reporting at least 1 event.

^b^Number of participants with at least 1 administered dose.

Within 30 days after vaccination, 130 participants reported a total of 208 unsolicited AEs (HZ-PreVac: 125 AEs in 78 participants; HZ-NonVac: 83 AEs in 52 participants) (Table 3). At least 1 grade 3 unsolicited symptom was reported by 14 participants in the HZ-PreVac group and 5 participants in the HZ-NonVac group. No evidence of clinically relevant differences in reported unsolicited AE was observed between study groups.

From study start until 30 days after the second vaccination, a total of 9 SAEs were reported in 8 study participants (HZ-PreVac: 5 SAEs in 4 participants; HZ-NonVac: 4 SAEs in 4 participants). None of these SAEs were considered related to vaccination by the study investigators. No deaths occurred, and no HZ cases or pIMDs were reported during the active phase of the study.

## DISCUSSION

This study showed that the humoral immune response to HZ/su 1 month post–dose 2 was noninferior in adults >65 years of age who were vaccinated with the live attenuated zoster vaccine (Zostavax) >5 years ago compared with adults who never received this vaccination. Moreover, HZ/su was well-tolerated in both study groups, and no safety concerns were identified from first vaccination up to 1 month post–dose 2.

As in previous studies, HZ/su induced robust immune responses [22–24]. This study showed that prior vaccination with ZVL does not negatively impact the humoral immune responses to HZ/su. In addition, descriptive analyses did not reveal any apparent differences in CMI responses as assessed by CD4^2+^ T-cell frequencies, and postvaccination increases in CD4^2+^ T cell-frequencies were observed in both study groups. Altogether, these observations suggest that vaccination with HZ/su may provide protection and therefore may be an attractive candidate to revaccinate adults >65 years of age who were vaccinated with ZVL >5 years ago.

Consistent with previous findings, HZ/su recipients frequently reported the occurrence of solicited local and systemic AEs. Reactogenicity, as observed during phase 2 and phase 3 clinical trials with HZ/su [22–24, 26, 27, 30–32], was characterized by transient injection site reactions, headache, fatigue, and myalgia. The findings in the study presented here show that reactogenicity after vaccination is not exacerbated in older adults who were previously vaccinated with ZVL. As such, reactogenicity in HZ/su recipients who previously received ZVL is unlikely to differ from ZVL-naive HZ/su recipients. Similarly, the number of SAEs reported were balanced between study groups, and no SAEs were considered related to vaccination by the study investigators. No fatal SAEs or pIMDs were reported in either study group from first vaccination up to 1 month post–dose 2.

Our findings need to be evaluated in consideration of the strengths and limitations of the study design. The sample size was calculated to demonstrate noninferiority of the humoral immune response with 99% power, and our matching strategy aimed to ensure that the selected baseline characteristics are similar in both groups. Nonetheless, matching resulted in a study population that was fully of white heritage. In addition, as the United States was one of the few countries where ZVL coverage was sufficiently high, only US study centers participated.

In sum, we show that following vaccination with HZ/su, the humoral response in adults who were previously vaccinated with the live attenuated zoster vaccine was noninferior to that in adults without any previous vaccination against HZ. Robust cellular immune responses were observed in both groups. No clinically significant differences in safety and reactogenicity were observed between the 2 study groups. Taken together, HZ/su was well tolerated and induced a strong immune response irrespective of prior vaccination with ZVL, and may therefore be an attractive option to revaccinate prior ZVL recipients.

## Supplementary Data

Supplementary materials are available at *The Journal of Infectious Diseases* online. Consisting of data provided by the authors to benefit the reader, the posted materials are not copyedited and are the sole responsibility of the authors, so questions or comments should be addressed to the corresponding author.

## Supplementary Material

Supplementary Table 1

Supplementary Table 2

Supplementary Table 3

Supplementary Data

## Notes

***Acknowledgments.*** The authors thank all study participants as well as all clinical investigators amongst whom: Marc Anderson (Tanner Clinic, Utah); Bruce Bowling (Regional Clinical Research, New York); Toby Briskin (Rapid Medical Research, Ohio); Maia Chakerian (Samaritan Center for Medical Research Medical Group, California); Christopher Chambers (Thomas Jefferson University, Pennsylvania); Michael Chen (The Corvallis Clinic, Oregon); Edmund Claxton (Maine Research Associates, Maine), Frank Eder (UHS Primary Care, New York); Charles Fogarty (Spartanburg Medical Research, South Carolina); Bradley Fox (Liberty Family Practice, Pennsylvania); Marshall Freedman (Columbia Medical Practice, Maryland); Steven Geller (Centennial Medical Group, Maryland); Stephen Halpern (Radiant Research, California); Paul Hartley (Preferred Primary Care Physicians, Pennsylvania); Casey Johnson (Johnson County Clinical Trials, Kansas); Katie Julien (J. Lewis Research Inc, Utah); Julia Kyle (Marshfield Clinic, Wisconsin); Richard Mills (PMG Research, South Carolina); Terry Poling (Heartland Research Associates, Kansas); Anthony Puopolo (Milford Emergency Associates, Massachusetts); George Raad (PMG Research, North Carolina); Jakkidi Reddy (Sierra Clinical Research, California); John Edward Teddy Witbeck (J. Lewis Research, Utah); and Jack Yakish (Westminster Family Medicine, Pennsylvania). The authors also thank the contributions of Estelle Berengier (GSK, Rixensart, Belgium); Grégory Catteau, Garry Edwards, and Brecht Geeraerts (GSK, Wavre, Belgium); Catherine Dettori and Olfa Guaddoudi (4Clinics on behalf of GSK, Wavre, Belgium); Ophélie Gascard (Keyrus Biopharma on behalf of GSK, Wavre, Belgium); Marjan Hezareh (Chiltern International on behalf of GSK, Wavre, Belgium); and Robynn Shannon (GSK, Pennsylvania). Medical writing services were provided by Jarno Jansen and Alpár Pöllnitz (XPE Pharma & Science on behalf of GSK). Editorial assistance and publication coordination were provided by Sara Blancquaert (XPE Pharma & Science on behalf of GSK).

***Author contributions.*** GSK takes a commitment to convey a message in a way that would be easily understandable by Health Care Professionals (supplement text). L. C., M. D., T. C. H., H. L., L. O., and I. V. conceived and designed the study. K. G., N. K., L. O., and P. J. collected or generated study data. L. C., K. G., N. K., H. L., and J. P. performed the study. L. C., M. D., K. G., T. C. H., N. K., H. L., L. O., and I. V. were involved in the analysis or interpretation of the data. All authors contributed to the writing/reviewing of the manuscript and approved the final version for submission.

***Financial support.*** This work was supported by GSK Biologicals SA, which was involved in all stages of the study conduct and analysis, and also took responsibility for all costs associated with the development and publishing of the present manuscript.

***Potential conflicts of interest.*** L. C., M. D., K. G., I. V., and L. O. are employed by the GSK group of companies. I. V. and L. O. own stock options as part of their employee remuneration. T. C. H. and H. L. were employed by the GSK group of companies at the time this study was designed and received their stock as part of employee remuneration. H. L. is currently employed by Pfizer Inc. T. C. H. is coinventor of the patent application related to the vaccine used in this study and is currently a consultant for the GSK group of companies. N. K. reports receiving grants from the GSK group of companies during the conduct of this study, as well as receiving grants from Merck & Co, Pfizer Inc, Sanofi Pasteur, MedImmune, Novartis, and Protein Science for work outside of this study. J. P. reports receiving principal investigator fees from the GSK group of companies during the conduct of the study. All authors have submitted the ICMJE Form for Disclosure of Potential Conflicts of Interest. Conflicts that the editors consider relevant to the content of the manuscript have been disclosed.
