# Supplementary material for: Immunogenicity and Safety of the HZ/su Adjuvanted Herpes Zoster Subunit Vaccine in Adults Previously Vaccinated With a Live Attenuated Herpes Zoster Vaccine
Source: J Infect Dis. 2017 Sep 20;216(11):1343–51. doi: 10.1093/infdis/jix482 (PMC5853346; doi:10.1093/infdis/jix482)
Supplement: Supplementary Table 1 [file jix482_suppl_supplementary_table_1.docx]

## Supplementary Table 1 – Distribution of matching criteria at vaccination

|  |  |  | HZ-NonVac | HZ-PreVac | |
| --- | --- | --- | --- | --- | --- |
|  |  |  | N=215 | N=215 | |
| Female | 65-69 | Immune-mediated diseases | 12 | 11 | |
|  |  | Diabetes mellitus | 10 | 15 | |
|  |  | Current depression |  |  | |
|  |  | Pulmonary disorders |  |  | |
|  |  | Heart conditions |  |  | |
|  |  | None of the above | 29 | 29 | |
|  | 70-79 | Immune-mediated diseases | 10 | 11 | |
|  |  | Diabetes mellitus | 16 | 11 | |
|  |  | Current depression | 8 | 7 | |
|  |  | Pulmonary disorders |  |  | |
|  |  | Heart conditions |  |  | |
|  |  | None of the above | 25 | 24 | |
|  | 80+ | Immune-mediated diseases | 1 | 0 | |
|  |  | Diabetes mellitus |  |  | |
|  |  | Current depression |  |  | |
|  |  | Pulmonary disorders |  |  | |
|  |  | Heart conditions |  |  | |
|  |  | None of the above | 0 | 1 | |
| Male | 65-69 | Immune-mediated diseases | 10 | 8 | |
|  |  | Diabetes mellitus | 10 | 9 | |
|  |  | Current depression |  |  | |
|  |  | Pulmonary disorders |  |  | |
|  |  | Heart conditions | 9 | 8 | |
|  |  | None of the above | 20 | 24 | |
|  | 70-79 | Immune-mediated diseases | 1 | 1 | |
|  |  | Diabetes mellitus | 15 | 17 | |
|  |  | Current depression |  |  | |
|  |  | Pulmonary disorders |  |  | |
|  |  | Heart conditions | 11 | 12 | |
|  |  | None of the above | 16 | 13 | |
|  | 80+ | Immune-mediated diseases |  |  | |
|  |  | Diabetes mellitus |  |  | |
|  |  | Current depression |  |  | |
|  |  | Pulmonary disorders |  |  | |
|  |  | Heart conditions | 6 | 8 | |
|  |  | None of the above | 6 | 6 |  |

HZ-NonVac = participants who never received the live-attenuated zoster vaccine (ZVL); HZ-PreVac = participants who received ZVL ≥5 years prior to study start. Participants were matched for age (65-69, 70-79, ≥80), Sex (male, female), Race (Caucasian, African American, Hispanic and Other) and medical condition (immune-mediated diseases, diabetes mellitus, current depression, pulmonary disorders, heart conditions, none of the above), allowing for 144 possible matching clusters. The matching clusters with the greatest number of screened adults were selected to reach the target enrollment number. Study participants in the HZ-PreVac group were matched to HZ-NonVac within each of these matching clusters. Of note is that all participants were of Caucasian ancestry (not shown in Table).
