# Supplementary material for: Immunogenicity and Safety of the HZ/su Adjuvanted Herpes Zoster Subunit Vaccine in Adults Previously Vaccinated With a Live Attenuated Herpes Zoster Vaccine
Source: J Infect Dis. 2017 Sep 20;216(11):1343–51. doi: 10.1093/infdis/jix482 (PMC5853346; doi:10.1093/infdis/jix482)
Supplement: Supplementary Table 2 [file jix482_suppl_supplementary_table_2.docx]

## Supplementary Table 2 – Pre-specified potential immune-mediated diseases (pIMDs)

| **Endocrine disorders** | **Liver disorders** | **Gastrointestinal disorders** |
| --- | --- | --- |
| Autoimmune thyroiditis (including Hashimoto thyroiditis)  Grave's or Basedow’s disease  Diabetes mellitus type I  Addison’s disease  Polyglandular autoimmune syndrome  Autoimmune hypophysitis | Autoimmune hepatitis  Primary biliary cirrhosis  Primary sclerosing cholangitis  Autoimmune cholangitis | Inflammatory Bowel disease, including Crohn’s disease, ulcerative colitis, microscopic colitis, ulcerative proctitis  Celiac disease  Autoimmune pancreatitis |
| **Musculoskeletal disorders** | **Vasculitides** | **Neuroinflammatory disorders** |
| Systemic lupus erythematosus and associated conditions  Systematic Scleroderma (Systematic sclerosis), including diffuse systemic form and CREST syndrome  Idiophatic inflammatory myopathies, including Dermatomyositis, Polymyositis  Antisynthetase syndrome  Rheumatoid arthritis and associated conditions, including Juvenile chronic arthritis and Still’s disease  Polymyalgia rheumatic  Spondyloarthritis, including ankylosing spondylitis, reactive arthritis (Reiter's Syndrome) and undifferentiated spondyloarthritis  Psoriatic arthropathy  Relapsing polychondritis  Mixed connective tissue disorder | Large vessels vasculitis including: giant cell arteritis such as Takayasu's arteritis and temporal arteritis.  Medium sized and/or small vessels vasculitis including: polyarteritis nodosa, Kawasaki's disease, microscopic polyangiitis, Wegener's granulomatosis, Churg–Strauss syndrome (allergic granulomatous angiitis), Buerger’s disease (thromboangiitis obliterans), necrotizing vasculitis and anti-neutrophil cytoplasmic antibody (ANCA) positive vasculitis (type unspecified), Henoch-Schonlein purpura, Behcet's syndrome, leukocytoclastic vasculitis. | Cranial nerve disorders, including paralyses/paresis (e.g. Bell’s palsy)  Optic neuritis  Multiple sclerosis  Transverse myelitis  Guillain-Barré syndrome, including Miller Fisher syndrome and other variants  Acute disseminated encephalomyelitis, including site specific variants: e.g. non-infectious encephalitis, encephalomyelitis, myelitis, myeloradiculoneuritis  Myasthenia gravis, including Lambert-Eaton myasthenic syndrome  Immune-mediated peripheral neuropathies and plexopathies, (including chronic inflammatory demyelinating polyneuropathy, multifocal motor neuropathy and polyneuropathies associated with monoclonal gammopathy).  Narcolepsy |
| **Skin disorders** | **Blood disorders** | **Others** |
| Psoriasis  Vitiligo  Erythema nodosum  Autoimmune bullous skin diseases (including pemphigus, pemphigoid and dermatitis herpetiformis)  Cutaneous lupus erythematosus  Alopecia areata  Lichen planus  Sweet’s syndrome  Localised Scleroderma (Morphoea) | Autoimmune hemolytic anemia  Autoimmune thrombocytopenia  Antiphospholipid syndrome  Pernicious anemia  Autoimmune aplastic anemia  Autoimmune neutropenia  Autoimmune pancytopenia | Autoimmune glomerulonephritis (including IgA nephropathy, glomerulonephritis rapidly progressive, membranous glomerulonephritis, membranoproliferative glomerulonephritis, and mesangioproliferative glomerulonephritis)  Ocular autoimmune diseases (including autoimmune uveitis and autoimmune retinopathy  Autoimmune myocarditis/cardiomyopathy  Sarcoidosis  Stevens-Johnson syndrome  Sjögren’s syndrome  Idiopathic pulmonary fibrosis  Goodpasture syndrome  Raynaud’s phenomenon |

CREST = Calcinosis, Raynaud's phenomenon, Esophageal dysmotility, Sclerodactyly and Telangiectasia (syndrome)

Study investigators were also instructed to use their professional judgment to determine whether a possible pIMD was auto-immune in origin and to record it as such
