# Supplementary material for: Immunogenicity and Safety of the HZ/su Adjuvanted Herpes Zoster Subunit Vaccine in Adults Previously Vaccinated With a Live Attenuated Herpes Zoster Vaccine
Source: J Infect Dis. 2017 Sep 20;216(11):1343–51. doi: 10.1093/infdis/jix482 (PMC5853346; doi:10.1093/infdis/jix482)
Supplement: Supplementary Table 3 [file jix482_suppl_supplementary_table_3.docx]

## Supplementary Table 3 – Frequency of gE-specific CD4^+^ T cells expressing at least two activation markers per 10^6^ cells and geometric mean concentrations of anti-gE antibodies

|  |  | **Cell-mediated immunity** | | | |  | **Humoral immunity** | | |
| --- | --- | --- | --- | --- | --- | --- | --- | --- | --- |
|  |  | **Frequency of CD4^2+^** | | | |  | **Anti-gE antibodies** | | |
| **Group** | **Timing** | **N** | **Q1** | **Median** | **Q3** |  | **N** | **GMC** | **95% CI** |
| **HZ-PreVac** | **Month 0 (pre-vac)** | 152 | 1.0 | 67.4 | 138.2 |  | 204 | 1784.3 | 1572.9–2024.1 |
|  | **Month 1 (post-D1)** | 177 | 240.6 | 425.1 | 673.0 |  | 204 | 29959.0 | 26633.6–33699.6 |
|  | **Month 3 (post D2)** | 170 | 1464.5 | 2312.1 | 4148.3 |  | 204 | 49327.2 | 45388.2–53608.1 |
| **HZ-NonVac** | **Month 0 (pre-vac)** | 140 | 1.0 | 58.1 | 160.3 |  | 202 | 1408.5 | 1203.3–1648.8 |
|  | **Month 1 (post-D1)** | 170 | 219.7 | 426.8 | 733.4 |  | 202 | 25233.7 | 22072.3–28848.0 |
|  | **Month 3 (post D2)** | 177 | 1448.6 | 2214.2 | 3734.5 |  | 204 | 51618.5 | 47224.8–56420.9 |

N = number of participants with available results; HZ-NonVac = participants who never received the live-attenuated zoster vaccine (ZVL); HZ-PreVac = participants who received ZVL ≥5 years prior to study start; 95% CI = 95% confidence interval; CD4^2+^, CD4^+^ T cells expressing at least two activation markers among CD40 ligand, interleukin-2, tumor necrosis factor-α, interferon–γ; GMC, geometric mean concentrations; Q1, Q3, first and third quartiles; pre-vac = before first dose of HZ/su; post D1 = one month after first dose of HZ/su; post D2 = one month after second dose of HZ/su
