# Supplementary material for: Immunogenicity and Safety of the HZ/su Adjuvanted Herpes Zoster Subunit Vaccine in Adults Previously Vaccinated With a Live Attenuated Herpes Zoster Vaccine
Source: J Infect Dis. 2017 Sep 20;216(11):1343–51. doi: 10.1093/infdis/jix482 (PMC5853346; doi:10.1093/infdis/jix482)
Supplement: Supplementary Data [file jix482_suppl_supplementary_data.docx]

# Focus on the Patient

What is the context?

Varicella-zoster virus (VZV), the virus that causes chickenpox, remains latent in the body after the initial infection. Reactivation of the virus can result in herpes zoster (or shingles), which is characterized by a painful, localized rash. The risk of shingles increases with age. Vaccination with the licensed live-attenuated zoster vaccine (ZVL) offers protection; however, this protection wanes over time. Therefore, vaccination of people who previously received ZVL with the new non-replicating candidate vaccine (HZ/su) may re-stimulate the immune response against VZV and provide protection against shingles.

What is new?

This clinical study was conducted to evaluate the immune response and safety of HZ/su vaccination in people that previously did or did not receive the ZVL at least 5 years earlier. The immune responses following HZ/su vaccination were robust in both groups of people. In both groups, the most common local reactions after vaccination were injection-site pain, redness and swelling. Fatigue, headache, and myalgia (muscle pain) were the most commonly observed general reactions. No safety concerns were identified in this study.

What is the impact?

This study showed that HZ/su vaccination induces strong immune responses in older adults who received the ZVL more than 5 years earlier and may help protect them against shingles.
